# Comparison of the effect of preheating on the flexural strength of giomer and nanohybrid composite resin

**DOI:** 10.34172/joddd.2022.027

**Published:** 2022-11-15

**Authors:** Soodabeh Kimyai, Zahra Mashayekhi, Narmin Mohammadi, Mahmoud Bahari, Mahdi Abed Kahnamouei, Mohammad Esmaeel Ebrahimi Chaharom

**Affiliations:** ^1^Dental and Periodontal Research Center, Faculty of Dentistry, Tabriz University of Medical Sciences, Tabriz, Iran; ^2^Department of Operative Dentistry, Faculty of Dentistry, Tabriz University of Medical Sciences, Tabriz, Iran

**Keywords:** Composite resins, Dental restoration, Flexural strength, Heating

## Abstract

**Background.** Considering the increased use of preheating and novel resin-based materials to restore teeth, the present study investigated the impact of preheating on the flexural strength of a giomer and compared it with a nanohybrid composite resin.

**Methods.** Two restorative materials (Beautifil II giomer and Alpha III nanohybrid composite resin) were used. Thirty rod-shaped samples (adding up to 60 samples) were prepared from the materials above and divided into two subgroups: with and without preheating (n=15). Before sample preparation, the giomer and nanohybrid composite resin tubes were preheated at 68ºC for 15 minutes in the preheating subgroups. In the subgroups without preheating, the tubes were kept in a room at 25ºC. Then the flexural strength was compared between the two groups with two-way ANOVA at a significance level of *P*<0.05.

**Results.** The results showed significantly higher flexural strength in the preheated subgroups than in the non-preheated subgroups (*P*<0.001). In addition, the mean flexural strength values were significantly higher in the giomer groups than in the nanohybrid composite resin groups (*P*<0.001).

**Conclusion.** Preheating increased the studied materials’ flexural strengths significantly. The flexural strength of the giomer restorative material was higher than that of the nanohybrid composite resin, irrespective of preheating.

## Introduction

 Currently, composite resins are the most widely restorative materials in dentistry. However, they have some limitations and should be improved. One of the techniques to improve resin-based materials’ properties is preheating, which is a simple, relatively successful method.^1^ It has been reported that preheating can increase the degree of conversion,^2^ improve marginal adaptation by decreasing viscosity,^3^ decrease gaps,^4^ and decrease polymerization shrinkage^5^ in some resin-based materials. In addition, according to previous reports, preheating can improve mechanical properties by increasing the polymerization of resin-based materials.^6^ However, sufficient evidence is not available on improving the quality and longevity of restorations after preheating.^1^ According to some studies, preheating composite resins does not affect some physical and mechanical properties of composite resins, including microhardness,^7^ degree of convergence conversion,^8^ polymerization stress,^8^ fracture toughness,^7^ marginal microleakage,^9^ color stability,^10^ and flexural strength.^6^

 Flexural strength is a mechanical characteristic of restorative materials and predicts the materials’ behavior under functional and parafunctional forces to some extent and can be an indicator of restorative materials’ clinical performance.^11^ A number of previous studies have investigated the restorative materials’ flexural strengths after preheating, with contradictory results,^2,3,6,12-14^ which have been attributed to differences in the formulation of the studied materials, the organic resin matrix, inorganic filler content, and the temperature and duration of preheating.^1,6^

 Sharafeddin et al investigated the flexural strength of nanohybrid and silorane-based composite resins after preheating and reported an improvement in the flexural strength after preheating to 45ºC.^12^ Deb et al studied the effect of preheating (at 60ºC) on the flexural strengths of five different types of composite resin and one compomer. They reported that only the flexural strengths of two composite resin types (i.e., Spectrum TPH hybrid and wave flow) increased, with no changes in other composite resins’ flexural strengths (i.e., Herculite Unidose XRV, Heliomolar, Filtek P60, F2000).^2^

 Uctasli et al reported no changes in the flexural strength of one nanohybrid and one microhybrid composite resin after preheating (40ºC, 45ºC, and 50ºC).^6^ In addition, according to Fróes-Salgado et al, preheating (68ºC) had no significant impact on the flexural strength of a nanofilled composite rein.^3^ Furthermore, Mohammadi et al reported that preheating (37ºC and 68ºC) did not affect the flexural strength of silorane-based and methacrylate-based composite resins.^13^

 D’Amario et al reported that a 20-round preheating procedure at 45ºC had no significant impact on the flexural strength of three different types of composite resin (two microhybrid composite resins and one nanofilled composite resin). However, a 40-round preheating procedure at 45ºC decreased the composite resins’ flexural strengths.^14^

 Giomers are novel light-cured nano-composite materials with pre-reacted glass-giomer fillers. These fillers are hydrogel silica particles that result from the reaction of fluoroaluminosilicate glass fillers with polyacrylic acid, cut into pieces and silanized after freeze-drying to produce filler particles to be incorporated into the resin matrix. These materials have the advantages of composite resins (superb esthetic appearance, easy polishing, and biocompatibility) and glass-ionomer (fluoride release and fluoride recharging capacity).^15,16^

 Dionysopoulos et al investigated the effect of preheating on Beautifil II giomer’s film thickness and concluded that preheating at 54ºC and 60ºC decreased the film thickness of the giomer.^17^ According to Dionysopoulos et al, preheating up to 54ºC increased the microhardness of Beautifil Bulk Restorative and Beautifil Bulk Flowable giomers.^18^

 Since preheating has diverse effects on the mechanical properties of resin-based restorative materials in terms of the type and composition of the material,^7^ and since no study has focused on the effect of preheating on the flexural strength of giomer, this in vitro study investigated the effect of preheating on the flexural strength of a giomer and compared it a nanohybrid composite resin.

## Methods

 This in vitro study included 30 rod-shaped samples (measuring 25 mm in length, 2 mm in width, and 2 mm in height)^12^ of the A3 shade of Beautifil II giomer (Shofu Dental Corporation, Osa­ka, Japan) and Alpha III nanohybrid composite resin (Dental Technologies, Inc., Lincolnwood, USA) restorative materials (n = 60). The Ethics Committee of Tabriz University of Medical Sciences, Tabriz, Iran, approved the study protocol.

 The sample size (n = 11 in each subgroup) was calculated based on a pilot study at α = 0.05, with a study power of 80% and a 9-unit difference in flexural strength means. However, the sample size was increased to n = 15 in each subgroup (adding up to 60 samples) to increase the study’s validity.

 Each restorative material’s samples were assigned to two subgroups (n = 15): with and without preheating. In the subgroup without preheating, the giomer and nanohybrid composite resin tubes were kept at ambient temperature (25ºC), and no preheating was carried out. In the preheated group, the giomer and nanohybrid composite resin tubes were immersed in a thermostatically controlled water bath (Teledyne Hanau, Buffalo, NY, USA) at 68ºC for 15 minutes.^1,13^ Then, the samples were prepared as follows.

 Rod-shaped samples (25, 2, and 2 mm in length, width, and height, respectively) were prepared from the two restorative materials using a silicone mold according to ISO 4049/2000,^12^ based on the manufacturer’s instructions. The materials were placed in each mold using a spatula and condensed with a condenser. A transparent matrix band (Hawe Neos Dental, Bioggio, Switzerland) was pressed on each mold using a glass slab to create a smooth surface. Then each sample was light-cured using a Dentamerica (San Jose Ave. Industry, CA 91748, USA) light-curing unit at 400 mW/cm^2^ light intensity perpendicular to the surface, barely touching it. After retrieving the samples from the molds, they were light-cured again for 20 seconds from each aspect to achieve complete polymerization. The samples were incubated in distilled water at 37ºC for 24 hours, followed by polishing the sample surfaces with medium, fine, and superfine polishing disks (Sof-Lex, 3M ESPE Dental Products St Paul, MN 55144-1000 USA). Then the samples were cleaned ultrasonically in distilled water for 1 minute.^11^

 The samples’ flexural strengths were determined using a universal testing machine (Hounsfield Test Equipment, Model HSK-S, Salfords, Redhill, Surrey, England) at a crosshead speed of 0.5 mm/min until fracture occurred. The flexural strength (σ) was calculated in MPa using the formula below^12^:

 σ = 3FL/2BH^2^

 where F is the force in Newton, L is the distance between the supports in mm (20 mm), and B and H were the samples’ width and height in mm, respectively.

 The data analyses were conducted with SPSS 17 (SPSS Inc., Chicago, IL, USA). The Kolmogorov-Smirnov test was applied to evaluate data normality. In addition, two-way analysis of variance (ANOVA) was used to evaluate the effects of preheating and restorative material type on flexural strength at a significance level of *P*<0.05.

## Results

**Figure 1:**
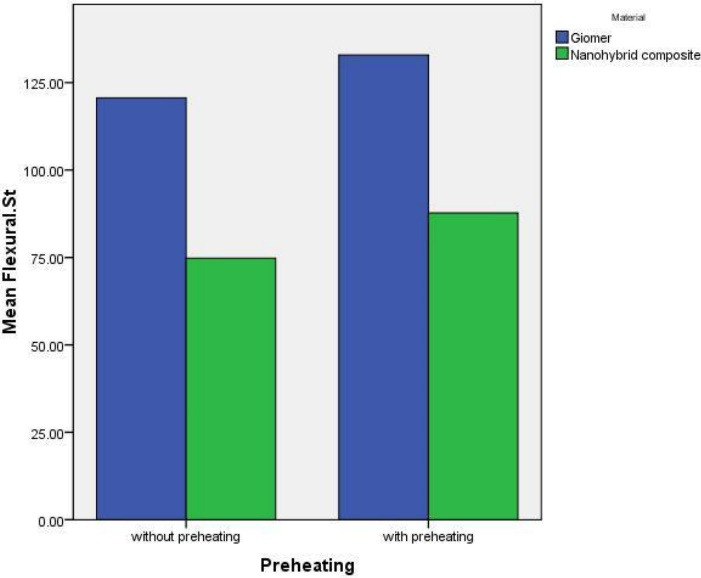
The error-bar graph of the mean flexural strengths in the study groups and subgroups in terms of preheating

Table 1 shows the descriptive statistics (means and standard deviations) of flexural strength values in different study groups and subgroups. Figure 1 is the error-bar graph of the mean flexural strength values in terms of preheating.

**Table 1:** The means and standard deviations (SD) of flexural strengths (MPa) in different study groups and subgroups

**Restorative material**	**Preheating**	**Mean±SD**	**No.**
Beautifil II giomer	Without preheating	120.67 ± 7.98	15
	With preheating	132.93 ± 10.26	15
Alpha III nanohybrid composite resin	Without preheating	74.79 ± 7.39	15
	With preheating	87.74 ± 9.30	15

 Two-way ANOVA revealed significantly higher flexural strengths in the preheated subgroups than in the subgroups with no preheating (F_1,56_ = 400.380, *P*<0.001).

 In addition, the mean flexural strengths in the giomer groups were significantly higher (F_1,56_ = 30.701, *P*<0.001). However, the cumulative effect of preheating and the restorative material type was not significant (F_1,56_ = 0.023, *P* = 0.881).

## Discussion

 Although many studies have investigated the effect of preheating on the efficacy of different restorative materials, sufficient data are not available on improvements in the quality and longevity of restorations after preheating restorative materials.^1^ The present study explored the effect of preheating on the flexural strength of a giomer and a nanohybrid composite resin. Three-point bending test was used to determine the materials’ flexural strengths. Three-point bending test has a lower variance coefficient, lower standard deviation, and lower crack propagation in dental composite resins compared to the biaxial bending test.^19,20^

 In the present study, preheating was carried out at 68ºC. A literature review showed that the mean preheating temperature is 54-68ºC, considered a safe temperature range because it does not result in pulpal irritation.^1^ Concerning the duration of preheating, despite a wide range of preheating times in different studies (from 40 seconds to 24 hours), the logical duration in clinical settings has been mentioned to be 15 minutes.^1^ Therefore, preheating was carried out for 15 minutes in the current study.

 In the present study, preheating increased flexural strength significantly, irrespective of the restorative material type. In this line, some previous studies have shown an increase in the flexural strength of resin restorative materials after preheating,^2,12^ which can be attributed to an increase in the degree of conversion in resin restorative materials. It has been reported that an increase in the monomer’s degree of conversion improves the physical and mechanical properties of composite resins.^21,22^ Preheating resin-based restorative materials can increase the monomer’s degree of conversion and increase the polymerization rate. An increase in the material’s temperature decreases its viscosity, increasing the motility of free radicals and propagation of polymer chains, which result in the completion of the polymerization reaction, formation of more double bonds, and increased cross-linking.^1,2,12^

 However, in contrast to the present study, some studies have reported that preheating did not affect the flexural strength of composite resins.^3,6,13^ The discrepancy in the results of the present study and some previous studies^3,6,13^ might be attributed to the type and formulation of the materials tested and their filler content and organic matrix.^1,7^ In addition, the preheating temperature might be another reason for differences between the present study findings and a study by Uctasli et al.^6^ In that study, preheating was carried out at 40ºC, 45ºC, and 50C, while in the present study, preheating was carried out at 68ºC. It has been reported that an increase in temperature might increase the monomer’s degree of conversion,^23^ improving composite resins’ physical and mechanical properties.^21,22^

 D’Amario et al reported that 20 rounds of preheating did not affect the flexural strength of composite resins; however, 40 rounds of preheating decreased it.^14^ The discrepancies between the present study and the above study might be explained by differences in the preheating process protocol. In the study above,^14^ preheating was repeated several times (20 and 40 rounds), while in the present study, it was carried out once.

 Another finding of the present study was that the flexural strength in the giomer groups was significantly higher than that in nanohybrid composite resin groups, irrespective of preheating.

 According to the manufacturer’s brochures, the flexural strength of Beautifil II giomer is 130 MPa, with 95 MPa for Alpha III nanohybrid composite resin. In the present study, too, in the subgroup without preheating, the mean flexural strength of giomer was higher than the nanohybrid composite resin (120.67 MPa vs. 74.79 MPa).

 The filler contents of giomer and nanohybrid composite resin are 83.3 and 64-84 wt%, respectively. According to previous studies, the inorganic filler content is directly related to the mechanical properties of restorative materials.^24^ An increase in filler content increases the flexural strength of composite resins.^25,26^ In the present study, the filler content had no relationship with the flexural strength of restorative materials; in contrast, the resin matrix type, polymerization kinetics, and fillers’ surface preparation affected the flexural strength of restorative materials.^25^

 In addition, it has been reported that there is a direct correlation between the size and distribution of fillers and the flexural characteristics of composite resins,^25,27^ which might explain a higher flexural strength in giomer than the nanohybrid composite resin irrespective of preheating.

 According to ISO 4049, the flexural strength in the three-point bending test of polymer-based restorative materials should be a minimum of 80 MPa.^22^ In the present study, in subgroups without preheating, the mean flexural strength of giomer was higher than 80 MPa, indicating that giomer can be safely used in areas under stress.^28^ It has been reported that composite resins with high flexural strength are less susceptible to bulk and marginal fractures.^29^

 Previous studies have not compared the flexural strengths of Beautifil II giomer and Alpha III nanohybrid composite resin. However, one study^29^ compared the flexural strength of Beautifil giomer (Shofu) and Alpha-Dent (Dental Technologies) microfilled hybrid composite resin, concluding that the flexural strength of Beautifil giomer was significantly higher than that of Alpha-Dent composite resin. It should be pointed out that the materials in the two studies were different despite similar results. Beautifil is the first generation of giomer restorative materials. However, Beautifil II (evaluated in the present study) belongs to the second generation of giomer resorptive materials, with improved properties.^30^ According to the brochures of the materials, Alpha-Dent is a microfilled hybrid composite resin, while Alpha III (evaluated in the present study) is a nanohybrid composite resin.

 In a previous study,^29^ the higher flexural strength of giomer compared to the microfilled hybrid composite resin was attributed to the higher filler content in giomer and the absence of HEMA (hydroxyl ethyl methacrylate) in the giomer structure. The absence of HEMA results in a decrease in water sorption. Extra water can serve as a plasticizer for resin, leading to hydrolytic damage to the filler and silane and decreased flexural strength of the material.^31^

 Ugurlu et al^32^ did not report significant differences in the flexural strength of Beautifil II and a nanofilled campsite resin (Estelite Sigma Quick) and a nanohybrid composite resin (reliaFIL LC) after 24 hours and one year. The discrepancy between the results of the present study and the study above might be attributed to the type and composition of the materials tested in the two studies.

 Restorative materials should have a high flexural strength to increase restoration longevity.^32^ Since the flexural strengths of giomer and nanohybrid composite resin increased after preheating, a preheating process is recommended under clinical conditions.

 Giomer can release fluoride ions preventing the demineralization of tooth structures.^33,34^ Considering the advantages above, this material’s application is on the increase. The present study evaluated the effect of preheating on this material’s flexural strength. It is suggested that future studies evaluate the effect of different preheating protocols (temperature, duration, and repeated preheating procedures) on other physical and mechanical characteristics of this material. In addition, long-term studies are suggested to evaluate the microstructural effects of preheating on restorative materials under an electron microscope.

## Conclusion

 It was concluded in the present study that preheating increased flexural strength irrespective of the material type. In addition, the giomer restorative material exhibited higher flexural strength than the nanohybrid composite resin with and without preheating.

## Acknowledgments

 The authors would like to thank Dr. Majid Abdolrahimi (D.D.S.), who edited the English text of this article.

## Author Contributions

 The study was planned by SK, NM, MB, and ZM. The literature review was performed by SK, ZM, NM, MB, MAK, and MEEC. SK and ZM performed the experiments and drafted the manuscript. The statistical analyses and interpretation of data were carried out by SK and NM. All the authors critically revised the manuscript for intellectual content. All the authors have read and approved the final manuscript.

## Funding

 The study was sponsored by the Vice Chancellor for Research at Tabriz University of Medical Sciences.

## Ethics Approval

 The study protocol was approved by the Ethics Committee at Tabriz University of Medical Sciences (Ref. No. IR.TBZMED.VCR.REC.1400.446).

## Competing Interests

 The authors declare no competing interests concerning the authorship and/or publication of this article.
